# The metric matters when assessing diversity: Assessing lepidopteran species richness and diversity in two habitats under different disturbance regimes

**DOI:** 10.1002/ece3.4581

**Published:** 2018-10-26

**Authors:** Lucinda Kirkpatrick, Sonia N. Mitchell, Kirsty J. Park

**Affiliations:** ^1^ Biological and Ecological Sciences University of Stirling Stirling UK; ^2^ EVECO, Universiteit Antwerpen Antwerp Belgium; ^3^ Boyd Orr Centre for Population and Ecosystem Health University of Glasgow Glasgow UK; ^4^ Institute of Biodiversity, Animal Health and Comparative Medicine, College of Medical, Veterinary and Life Sciences University of Glasgow Glasgow UK

**Keywords:** anthropogenic disturbance, functional diversity, habitat management, Lepidoptera

## Abstract

How we measure diversity can have important implications for understanding the impacts of anthropogenic pressure on ecosystem processes and functioning. Functional diversity quantifies the range and relative abundance of functional traits within a given community and, as such, may provide a more mechanistic understanding of ecosystems. Here, we use a novel approach to examine how lepidopteran richness and diversity, weighted by species abundance, differ between habitats under different disturbance regimes (highly disturbed non‐native plantations and less disturbed broadleaf woodlands), both with and without constraining by similarity due to shared taxonomy or functional traits. Comparisons of diversity between the two habitats differed according to which metric was being used; while species richness was 58% greater in broadleaf woodlands, after accounting for species similarity due to shared functional traits, there was little difference between woodland types under two different disturbance regimes. Functional diversity varied within the landscape but was similar in paired broadleaf and plantation sites, suggesting that landscape rather than local factors drive biotic homogenization in plantation dominated landscapes. The higher richness in broadleaf sites appears to be driven by rare species, which share functional traits with more common species. Moth populations in disturbed, plantation sites represent a reduced subset of moth species compared to broadleaf sites, and may be more vulnerable to disturbance pressures such as clear‐felling operations due to low community resilience.

## 1 INTRODUCTION

Widespread concerns about the impact of human activities on ecosystems have made the accurate measurement and assessment of biodiversity increasingly important. Species richness has long been the most commonly employed measure of biological diversity, stemming from the premise that species delineations are distinct, but this can be contentious (Hooper et al., 2002). Additionally, in most indices of species diversity all species are treated as equally different from each other, whereas this is clearly not the case (Chao, Chiu, & Jost, 2014; Leinster & Cobbold, 2012). For example, a community comprising distantly related species has more evolutionary diversity than a community with only closely related species. Likewise, an assemblage in which species share similar functional traits is less diverse than an assemblage with a range of functional traits, and may correspondingly result in reduced ecosystem functioning.

Measures of functional diversity quantify the range and relative abundance of particular functional traits within a community, offering a more mechanistic understanding of ecosystems than that obtained from simple patterns of species diversity and evenness (Hooper et al., 2002). As such, they can inform ecosystem managers about the impacts of anthropogenic pressures on the suite of functional traits in a community, and the consequential effects on ecosystem processes (Hooper et al., 2005; Tilman, 2000). While species richness may act as a suitable surrogate for functional diversity, this will depend on patterns of species assemblage. In the absence of environmental filtering (the process by which abiotic factors limit the establishment or persistence of species with particular functional traits in a particular location), functional diversity may be expected to increase linearly with species richness. However, in systems characterized by disturbance and recovery, environmental filtering may result in reduced functional richness compared to species richness as specific functional traits could be disproportionately unable to persist through disturbance (Mori, Furukawa, & Sasaki, 2013). Therefore, relying solely on metrics such as species richness can be misleading when attempting to assess the impacts of disturbance on an ecosystem. Furthermore, while the loss of a single species is serious from a conservation perspective, from a functional perspective a resilient ecosystem with high functional redundancy will be sustained through environmental perturbations despite loss of individual species (Mori et al., 2013). Measures of functional redundancy and diversity can therefore be used to assess the vulnerability of ecosystems to changes of state—from a functioning system to one with a reduced number of ecosystem services (Mori et al., 2013)—when species richness or indices of diversity such as Shannon's entropy may not detect the loss of key functional traits.

Here, we use moths, a key component of terrestrial ecosystems, to explore how the impact of disturbance on a community differs between different metrics of species richness. We compare moth populations in native broadleaf woodlands, under a minimal disturbance regime, with intensively managed conifer plantations. Moths perform a variety of roles within ecosystems as herbivores, pollinators, and prey species for a range of taxa such as birds, small mammals, and bats (Fox, Parsons, & Chapman, 2013). In recent decades, they have undergone substantial declines; two thirds of common and widespread species have suffered rapid population decreases in the United Kingdom (Conrad, Warren, Fox, Parsons, & Woiwod, 2006), with similar patterns occurring elsewhere in Europe (Franzén & Johannesson, 2007; Mattila, Kaitala, Komonen, Kotiaho, & Päivinen, 2006). Rapid economic development, urbanization, agricultural expansion, and changes to silvicultural practices have all been implicated (Conrad et al., 2006; Fox et al., 2013), although there is little information on the impacts of forestry management on moth communities in temperate plantations (but see Kirkpatrick, Bailey, & Park, 2017). Many plantation forests are even‐aged, with a simplified structure resulting from the loss of horizontal (spatial heterogeneity) and vertical (stratification) structural diversity (Sullivan, Sullivan, Lindgren, & Ransome, 2009). This is likely to support a lower invertebrate diversity than native or uneven‐aged forests due to a lack of old growth conditions and suitable understory habitat for a variety of species (Sullivan et al., 2009).

Despite the suggestion that afforestation with non‐native conifers is a key driver of moth declines, there is little information on how moth communities in coniferous plantations compare to those in more favorable habitats such as native broadleaved woodlands. In addition, the role functional diversity may play in determining moth community compositional differences is unclear. In this study, we aimed to determine the following:

1. How does the metric of diversity (species richness and diversity compared to functional richness and diversity) alter the quantification of diversity in habitats under differing disturbance regimes?
2. Does the level of redundancy (i.e., multiple species filling the same functional niche) differ between habitats under differing disturbance regimes?
3. Is environmental filtering occurring in habitats under differing disturbance regimes?

## 2 METHODS

Semi‐natural broadleaf woodlands and paired plantation sites were surveyed in over an 8 week period in 2014 (mid June until mid August) and a 7 week period in 2015 (early June until late July). Thirteen pairs of broadleaved and plantation woodland sites were selected in Galloway Forest Park, South West Scotland (Supporting Information Appendix S1: Figure S1, total sites = 26). Before planting, much of the Galloway area consisted of open upland and moorland habitat with low deciduous woodland cover due to historical deforestation. For comparison with plantations, we identified broadleaved woodlands that were well established (since at least 1840), all of which were over 20 ha in size. Broadleaf sites were either owned by Forestry Commission, or non‐governmental organizations; all were managed for biodiversity targets, but active management was minimal. Whilst the previous disturbance histories of these sites could not be determined, there had been no recent felling activity within the patches (which has been shown to detrimentally impact moth species richness if dominant in the landscape; Kirkpatrick et al., 2017). Plantations consisted of a mosaic of felled areas adjacent to mature stands (previous work in the same area suggests that there is little difference in moth richness and diversity between different stand types; Kirkpatrick et al., 2017). Plantation and broadleaf pairs were a mean 3.5 km (*SD* = 2.4) apart, and at a similar altitude.

### 2.1 Invertebrate sampling protocol

Broadleaf and plantation sites were surveyed for a single night, using a paired design. Site pairs were surveyed within 7 days of each other, so we assume that differences in the composition of paired sites is due to site differences rather than sampling date differences, and we randomized the order in which either broadleaf or plantation sites were surveyed first. We recognize that this only provides a snapshot of the moth species present at that time. However, here we are primarily interested in comparing moth communities and their functional traits between habitats rather than documenting the entire suite of species at each site. This method has been used previously to identify the impacts of harvesting on moths in commercial plantations (Kirkpatrick et al., 2017). Moths were trapped using portable 6W heath light traps using E7586 9″ actinic tube lights, powered with 12 V batteries which were activated 15 min after sunset and switched off after 4 hr (approximating the duration of the shortest night in the study area). This ensured that species flying at dusk and night were surveyed regardless of night duration. Species flying at dawn would most likely be missed, as traps were often turned off before dawn. Nights were only surveyed that were above 8°C in temperature with wind speeds less than Beaufort 4. Within each site two heath traps were used, placed 30 m from the wood edge and at least 50 m from each other, and results from both traps were pooled. In plantation sites, one heath trap was placed in a felled stand and another placed in a mature stand 5 m from the edge. Traps were positioned in such a way that the light was not visible from one trap to another. At the end of the surveying period, any moths attached to the outside of the trap were gently removed and released. A cotton wool ball soaked in ethyl acetate was immediately added to the trap and left overnight to kill trapped invertebrates. Macro moths were removed and pinned to boards for later identification by consultation with local recorders, and although micro moths were also separated for identification by an expert they are not included in this study as there is insufficient information available about their functional traits.

### 2.2 Functional trait identification

To understand the variation in functional richness and diversity of moth species in both plantation and broadleaf woodlands, we selected six traits that have been previously identified as potential predictors of moth extinction (Franzén & Johannesson, 2007). Of these, the moths sampled only showed sufficient variation for analysis in four traits (see Supporting Information Appendix S1: Table S1): larval host plant preference, larval specialism (whether the larvae specialized on a single plant family or multiple families), overwintering stage (egg, cocoon, pupa, N/A), and wingspan (tentatively linked with dispersal ability, Sekar, 2012). Trait values were obtained from Waring and Townsend (2009). Here we focus on between‐species variation, as traits were largely categorical, and we had a small sample set for measured traits.

All analyses were carried out in R (R core development team) using the following packages: FD, vegan, and rdiversity. To quantify how the moth community functional richness and diversity differed between broadleaf and plantation sites, we used the rdiversity package, which extends Hill numbers (Hill, 1973) to incorporate similarity, for example, taxonomic, phylogenetic, functional, etc. (Leinster & Cobbold, 2012; Reeve et al., 2016).

### 2.3 Calculating diversity measures

Hill numbers are a family of measures that unify many traditional indices of diversity and are expressed as the effective number of species present in a population (Hill, 1973). This framework is written,

$${}^{q}D\left(p\right)=M_{1-q}\left(p,p^{-1}\right)$$

which is the (1 − *q*)th order power mean of *p*, weighted by the inverse of *p*, and *p* = {*p*_1_, …, *p_S_*} denotes the relative abundance of species in a population for $\sum_{i=1}^{S}p_{i}=1$, where *p_i_* is the relative abundance of the *i*th species and *S* is the total number of species. Hill defines a parameter, *q*, which varies the sensitivity toward species rarity, where *q* = 0, 1, and 2 are equivalent to species richness, Shannon diversity, and Simpson diversity, respectively. Plotting these values as a diversity profile allows visual assessment of ecosystem or community diversity from a range of perspectives.

In recognition of the fact that species are not always equally different, Leinster and Cobbold (2012) extended Hill numbers to incorporate similarity, written:

$${}^{q}D^{Z}\left(p\right)=M_{1-q}\left(p,{\left(\mathrm{Zp}\right)}^{-1}\right)$$

where *Z* is an *S* × *S* similarity matrix comprising elements that take values between 0 and 1, such that species are completely identical when $Z_{ii^{\prime }}=1$ and completely distinct when $Z_{ii^{\prime }}=1$. Like Hill numbers, similarity‐sensitive diversities are expressed as effective numbers and can therefore be compared across multiple populations and represented graphically as a diversity profile.

Here, we term situations where all species are assumed to be equally different as “naïve” in contrast to similarity‐sensitive measures “taxonomic” (taxonomic similarity incorporated) or “functional” (similarity due to functional trait values included) richness and diversity.

rdiversity was used to calculate similarity‐sensitive diversity, requiring the construction of a normalized abundance matrix and a similarity matrix. We used the “gowdis” function in the R package “FD” to generate functional similarity matrices. A similarity matrix was constructed for each functional trait separately (see Supporting Information Appendix S1: Table S2A–C for examples of unconstrained (naïve), taxonomic, and functional similarity matrices). Since the phylogenetic tree for moths is insufficiently resolved to incorporate phylogenetic similarity, we constructed taxonomic similarity matrices by determining the taxonomic rank of each species, and using the “taxa2dist” function in the R package “vegan” to create a taxonomic similarity matrix. Species which are in the same genus or family will be more similar than species in different families (see Supporting Information Appendix S1: Table S2 for an example of a taxonomic similarity matrix). Population abundance matrices were normalized before further analysis in rdiversity. Changes in functional redundancy were calculated as the ratio of naïve species richness (i.e., where *q* = 0) to species richness constrained by functional similarity:

$$\mathrm{functional}\;\mathrm{redundancy}=\frac{\mathrm{na}\ddot{i}\mathrm{ve}\;\mathrm{species}\;\mathrm{richness}}{\mathrm{functional}\;\mathrm{species}\;\mathrm{richness}}$$

### 2.4 Statistical analysis

Differences in naïve, taxonomic and functional richness, diversity, evenness (objective 1) and redundancy (objective 2) for a range of traits (see Supporting Information Appendix S1: Table S1 for details) between plantation and broadleaf woodlands were tested using generalized linear mixed effects models with a Poisson error distribution (for “naive” measures) and a linear mixed effect model with a Gaussian error distribution (for “constrained” measures). Site nested in year and site nested in month were included as random effects to account for the paired design and the fact that different site pairs were sampled in 2 years and in different months. Models were validated by visual assessment of the residuals (Crawley, 2007). The conditional *R* ^2^ (variance explained by both the fixed and the random effects; Nakagawa & Schielzeth, 2013) was used to assess the amount of variation explained by each model. Explanatory variables were considered to have a “significant” effect on the responses if the standard error of the estimate did not cross zero.

We used a null model approach to test whether community composition in broadleaf and plantation woodlands showed evidence of environmental filtering (objective 3; Crawley, 2007). Null models allow the comparison of the observed communities with randomly assembled communities of equal species richness (Swenson, 2014). To achieve this, we randomly permuted (*n = *999) moth abundance across each site we sampled. For each randomization we calculated functional diversity measures, using the standardized effect size (SES) to compare the deviation of observed values relative to the null model assemblage (Rolo, Rivest, Lorente, Kattge, & Moreno, 2016). The SES is calculated as the ratio of the difference between the observed value and the mean of the null distribution to the standard deviation of the null distribution. The null hypothesis is that the average SES is zero; significantly higher values indicate niche complementarity, in which species coexist by using resources differently, whereas lower values indicate environmental filtering. We used linear models, excluding the intercept, to determine whether mean ± *SE*. SES values significantly deviated from zero.

## 3 RESULTS

### 3.1 Difference in naïve, taxonomic and functional measures of diversity between sites with differing disturbance regimes

**Figure 1:**
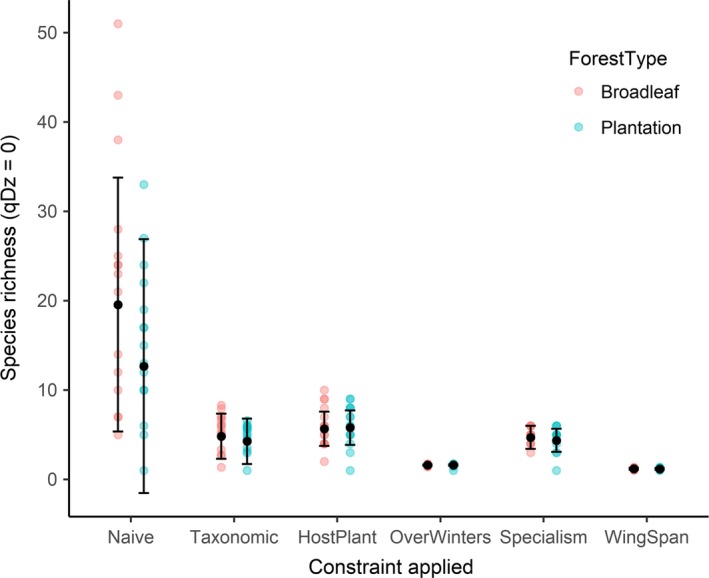
The difference between broadleaf and plantation sites for naïve, taxonomically and functionally constrained species richness. Colored dots depict broadleaf and plantation sites, with the point color darker where points overlap. Black points are the model predictions with error bars showing the standard errors

Naïve species richness in plantations was only 42% of that recorded in broadleaf woodlands; a mean of 27 (±5) compared to 63 (±3) species, respectively. However, after constraining for taxonomic and functional similarity, species richness was similar between woodland types (Table 1, Figure 1). Similarly, we found no difference in either Shannon (*q = *1) or Simpson's (*q = *2) diversity between plantation and broadleaved sites after constraining for functional similarity (Table 1), suggesting that diversity in both plantation and broadleaf woodlands is driven by the presence of rare individuals but does not differ significantly between the two habitat types (Table 1). The conditional *R* ^2^ for functional richness and diversity constrained by similarity due to host plant preferences was high, indicating greater variation between site pairs than woodland type per se (Table 1). However, for others, such as species richness constrained by wingspan, there was no difference between sites or habitat types (Table 1). When only the more common species were considered (*q* = 2), differences between site pairs in wingspan and overwintering style were more visible (Table 1).

**Table 1:** Best approximating GLMM's assessing the difference between paired broadleaved and plantation sites for naïve and constrained measures of species richness, diversity, dominance, and functional redundancy

Alpha diversity measures	Constraint	Intercept (Broadleaf woodland)	Plantation	*T*‐value	Marginal	Conditional
					*R* ^2^	*R* ^2^
Species richness (gaussian/poisson) (*q* = 0)	Naïve	2.5 ± 0.3	**−0.4 ± 0.1**	−3.9	0.06	0.67
	Taxonomic	4.8 ± 0.4	−0.6 ± 0.4	−0.9	0.02	0.76
	Host plant	5.7 ± 0.6	0.1 ± 0.5	0.2	0.01	0.41
	Larval specialism	4.6 ± 0.9	−0.3 ± 0.3	−0.9	0.01	0.52
	Overwintering stage	1.2 ± 0.1	0.0 ± 0.0	0.4	0.00	0.53
	Wing span	1.2 ± 0.0	0.0 ± 0.0	−0.4	0.01	0.08
Shannon diversity (gaussian) (^1^D, *q* = 1)	Naïve	18.5 ± 6.5	**−6.3 ± 3.0**	−2.1	0.06	0.65
	Taxonomic	3.6 ± 0.8	−0.1 ± 0.3	−0.4	0.00	0.77
	Host plant	4.4 ± 0.8	0.3 ± 0.4	0.7	0.01	0.64
	Larval specialism	4.1 ± 0.5	−0.3 ± 0.3	−1.1	0.02	0.44
	Overwintering stage	1.6 ± 0.0	0.0 ± 0.1	0.0	0.02	0.44
	Wing span	1.2 ± 0.0	0.0 ± 0.0	−0.4	0.01	0.53
Simpson diversity (gaussian) (^2^D, *q* = 2)	Naïve	17.4 ± 6.0	**−5.7 ± 2.8**	−2.0	0.05	0.64
	Taxonomic	3.6 ± 0.3	0.0 ± 0.3	0.1	0.00	0.00
	Host plant	3.8 ± 0.8	0.3 ± 0.4	1.3	0.00	0.67
	Larval specialism	3.7 ± 0.4	−0.3 ± 0.3	−0.8	0.01	0.46
	Overwintering stage	1.6 ± 0.0	0.0 ± 0.0	0.1	0.00	0.12
	Wing span	1.2 ± 0.0	0.0 ± 0.0	−0.4	0.00	0.53
Functional and taxonomic redundancy (gaussian) (*q* = 0)	Taxonomic	3.8 ± 0.6	**−0.8 ± 0.4**	−1.8	0.08	0.28
	Host plant	3.3 ± 0.9	**−1.2 ± 0.4**	**−3.1**	0.12	0.66
	Larval specialism	4.1 ± 0.9	**−1.2 ± 0.6**	−1.9	0.08	0.43
	Overwintering stage	12.3 ± 4.0	**−4.5 ± 1.9**	**−2.3**	0.07	0.63
	Wing span	15.9 ± 5.4	**−5.3 ± 2.7**	**−2.0**	0.05	0.61

Note

Parameters in bold are those which have a significant effect on response values, determined by whether the standard error of the estimate crosses zero (Burnham and Anderson). Marginal (*R* ^2^ explained by fixed effects) and conditional (*R* ^2^ explained by both fixed and random effects) as calculated by (Nakagawa & Schielzeth, 2013) presented.

### 3.2 Differences in functional redundancy between broadleaf and woodland sites

**Figure 2:**
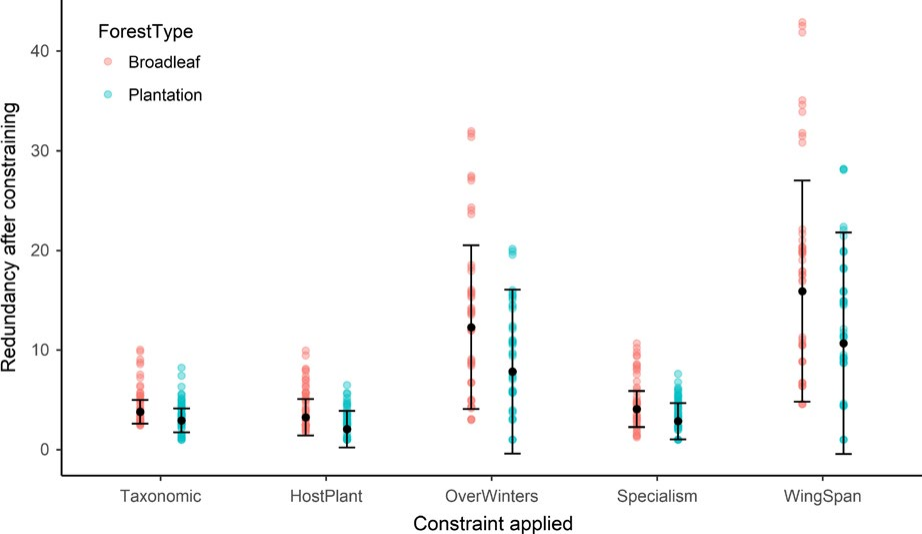
Difference in taxonomic and functional redundancy (the ratio of naïve species richness (i.e., where *q* = 0) to the constrained species richness (redundancy = naïve SR/functional SR) between broadleaf and plantation sites. Colored dots depict raw data for broadleaf and plantation sites, with the point color darker where points overlap. Black points are the model predictions with error bars showing the standard errors

Broadleaf woodland sites had greater functional redundancy than plantations with broadleaf woodlands containing a mean 9.3 (7.0 ± 2.4) species sharing similar host plant preferences, compared to 6.4 (±2.3) in plantations (Figure 2). Similarly, species sharing overwintering styles were 30% more numerous in broadleaf woodlands compared to plantations (Table 1, Figure 2). Finally, broadleaf woodlands had significantly more species with a greater range of wingspan sizes than plantations, although average wing length did not differ between broadleaf and plantation sites (ANOVA, *df* = 27, *F* = 0.12, *p* = 0.72). There appeared to be little difference in larval specialism or taxonomic diversity between broadleaf and plantation sites (Table 1).

### 3.3 Environmental filtering in habitats under differing disturbance regimes

We found no evidence that functional richness or diversity deviated significantly from zero for either broadleaf or plantation paired sites (Table 2), indicating no signal of environmental filtering or niche complementarity occurring in broadleaf compared to plantation sites.

**Table 2:** Standardized effect sizes (SES ± *SE*) and *p* values regressed against per habitat type of ancient semi‐natural broadleaf woodland or plantation woodland for all trait values as compared to a null model

Constraint	Broadleaf woodland		Plantation	
	SES	*p* Value	SES	*p* Value
Host plant	0.1 ± 0.3	0.6	−0.3 ± 0.3	0.3
Larval specialism	−0.3 ± 0.2	0.2	0.3 ± 0.2	0.1
Overwintering stage	0.2 ± 0.5	0.8	−0.1 ± 0.5	0.8
Wing span	0.0 ± 0.2	0.8	0.2 ± 0.2	0.3

## 4 DISCUSSION

This study demonstrates that non‐native coniferous plantations harbor similar moth functional diversity to native broadleaf woodlands, but the level of functional redundancy in plantations is lower, particularly for certain functional traits. Furthermore, we show that the metric used to assess diversity can result in differing interpretations of how diverse two contrasting habitats are, emphasizing the importance of care when selecting diversity metrics. This is particularly pertinent as many studies rely on single indices of diversity (e.g., Shannon Index and species richness), whereas this approach provides a more complete assessment of diversity.

### 4.1 Using naïve and constrained diversity indices to measure diversity

How best to limit and mitigate diversity loss due to anthropogenic practices is the key question for conservation (Sutherland et al., 2006). Research on the impacts of environmental change on diversity traditionally adopt a “naïve” approach, by focussing on the diversity of particular taxonomic groups without taking into account similarities between species due to functional, genetic or phylogenetic relationships (Spake, Barsoum, Newton, & Doncaster, 2016). However, the usefulness of these approaches will depend on the species assemblages (Chiu & Chao, 2014) and provides little information on mechanistic links between taxa and their environment (Hooper et al., 2002). Using indices developed by Leinster and Cobbold (2012), whilst incorporating functional or taxonomic similarity, it is possible to determine whether rare or abundant species are driving different patterns in functional diversity. These results are expressed as “effective numbers”, which permit an intuitive and clear comparison of differences in diversity in different habitat types.

### 4.2 Differences in diversity between native broadleaf woodlands and non‐native coniferous plantations

The size and proximity of broadleaf patches surrounding and within plantation stands has a marked positive effect on species richness (Kirkpatrick et al., 2017) so it was expected that functional richness and diversity would be higher in broadleaved woodlands. Surprisingly, although naïve richness was lower in plantation sites compared to broadleaved sites, we found no difference in functional richness or diversity. This contrasts with findings from other studies; in native woodlands managed for logging, particular functional guilds were negatively impacted by felling, and were therefore less diverse than in unmanaged woodlands (Summerville & Crist, 2002) whereas in highly urbanized parks butterfly functional diversity was lower than in parks with a less central location (Lizée, Mauffrey, Tatoni, & Deschamps‐Cottin, 2011). However, plantations differ from native woodlands in that open specialist species may persist in early successional stands produced during felling cycles (Spake et al., 2016), increasing the functional diversity in plantation sites. Furthermore, some lepidopteran species may be able to disperse from remnant patches of broadleaf maintained within the plantation landscape (Kirkpatrick et al., 2017). An alternative explanation for the somewhat surprising similarities between our plantation and broadleaf sites may be that the impact of intensive non‐native conifer management may act equally on moth communities in both plantation and broadleaf sites, with the extent of the impact mitigated by the surrounding landscape composition.

The lack of functional diversity differences between broadleaf and plantation sites may also reflect historical species loss that has not been offset by post‐harvest recovery of the original species assemblages (Summerville, 2015), particularly as harvesting is occurring on a constant cycle within the plantation landscape. Before planting the landscape consisted predominantly of open moorland, therefore, connectivity between broadleaf patches may still have been low. Unfortunately, we do not have information about lepidopteran species assemblages in broadleaf patches prior to the initial planting which would shed further light on whether biotic homogenization has occurred as a result of widespread non‐native conifer planting. Composition of the lowest diversity stands was dominated by species utilizing shrubs and trees as hosts during larval stages, which almost exclusively consisted of polyphagus species which feed on a range of common woody tree species. In contrast the moth community composition in more functionally diverse sites consisted of both monophagus and polyphagus species, including many herb or tree specialists (species which feed on a single herb or tree family during the larval stage). Greater specialization in host plant preferences is likely to render lepidopteran species vulnerable to disturbance (Summerville, 2011), and are therefore less likely to persist in heavily disturbed landscapes. The most functionally diverse paired sites also included a number of lichen specialist feeders and detritivores, which are often limited in distribution due to specific requirements for soil moisture and temperature (Bommarco et al., 2010). Therefore, not only was there a greater range of host plant preferences in the more diverse sites, but there was also a greater number of moth species that specialize on a single tree or plant family. Somewhat surprisingly, despite the predominance of conifer in the landscape, we did not find a large number of conifer tree specialists, even in functionally poor sites. This may reflect the unsuitability of Sitka spruce as a host tree for native moth species, even conifer specialists.

In general, areas with low functional diversity for traits related to feeding contained a high functional diversity when considering overwintering behavior. Areas with lower functional diversity were dominated by species which overwinter as eggs, rather than pupae, adults, and larvae, all of which were more represented in areas with higher functional diversity in traits associated with feeding. Species which overwinter as an adult or a pupa can move to find resources after emergence, which may represent an advantageous foraging strategy in response to landscape intensification (Lizée et al., 2011). Therefore, we would expect to find sites with low functional diversity for feeding behaviour to also harbor species which overwinter as adults or pupa, rather than eggs. High caterpillar mortality has been associated with mowing in grasslands; the occurrence of particular moth species will also relate to the survival of their larvae in response to land management practices such as harvesting (Mangels, Fiedler, Schneider, & Bluthgen, 2017). As non‐target trees are retained during felling, species which overwinter as eggs on trees rather than pupa or larvae underground may be less impacted by felling practices.

Previous work found that naïve moth richness and diversity in commercial coniferous plantations was influenced more by landscape than local factors (Kirkpatrick et al., 2017). As we used a paired design that restricted the distance between paired sites, landscapes surrounding the woodland patches surveyed here were intentionally similar, therefore, if differences between broadleaf and plantation sites were due to the local habitat, this should have been visible. The high conditional *R* ^2^ (variance explained by both random, i.e., “site pair” and fixed factors compared to fixed factors alone; Nakagawa & Schielzeth, 2013), for some models suggests that pair specific landscape factors may play a more important role than differences between broadleaf and plantation sites per se. It should be noted that including pair nested in month (to account for differences in sampling time) had a negligible impact on the *R* ^2^, suggesting that functional diversity differed more between site pairs than it did between sampling periods. In old growth forests with limited anthropogenic impacts, Summerville, Courard‐Hauri, and Dupont (2009) found that patterns of regional and local dominance were similar in stands recovering from logging. Species at logged sites were also disproportionately generalists; this signal of disturbance persisted for over 60 years, suggesting that the impacts of timber felling on lepidopteran communities are manifest over long time spans (Summerville et al., 2009). The presence of generalist species in more “disturbed” or intensively managed areas is common from a number of studies investigating functional diversity in a range of habitat types (Lizée et al., 2011; Mangels et al., 2017; Summerville et al., 2009). Many of the broadleaf patches in our study were relatively small and embedded within the plantation matrix. Therefore, it is possible that landscape management features may act as a filter on potential colonizing species, also negatively impacting broadleaf patches within the plantation landscape (Lizée et al., 2011). Site pair level differences may reflect a high preponderance of felling in the surrounding landscape, resulting in disturbed lepidopteran communities and local extinction of species with specific functional attributes in both plantation and broadleaf sites. The biotic homogenization and functional loss in both broadleaf and plantation stands in certain areas of the plantation dominated landscape mirrors that were found in areas of agricultural (Gámez‐Virués et al., 2015) and urban (Lizée et al., 2011) intensification. Evidence from other arthropod species suggests that the landscape can act as a strong filter for biotic traits (Gámez‐Virués et al., 2015); although we find no evidence of environmental filtering based on habitat type per se, it is likely that filtering is occurring in response to some other landscape variable, for example, the amount and intensity of logging in the surrounding area (Gámez‐Virués et al., 2015; Lizée et al., 2011). It would therefore be interesting to investigate how the intensity of plantation management impacts lepidopteran functional diversity, to further understand the mechanisms driving functional diversity loss in Lepidoptera.

There was no evidence of environmental filtering or niche complementarity occurring between plantations and broadleaf sites, as the SES did not differ significantly from zero. We did, however, find lower functional redundancy in plantations, particularly after constraining for similarity in host plant preference, overwintering stage and wing span. Potentially, moth populations in plantations represent a reduced subset of moth populations compared to the surrounding area, and may be more vulnerable to disturbance pressures such as felling due to low community resilience (Elmqvist & Folke, 2003; Soga et al., 2015). However, the mechanism behind the lower number of species is not clear, as lower redundancy was seen across all functional groups (Mattila et al., 2006).

## 5 CONCLUSIONS

Choosing which metric to use to assess diversity is important. We used a novel approach to measure diversity after constraining both for species abundance and species similarity. Using this approach allows a more detailed but also more rounded assessment of diversity, which can detect patterns which may not be immediately obvious if alternative approaches are used. For example, although naïve richness and diversity was significantly lower in habitats characterized by an intense disturbance regime, when we constrain by functional similarity there appears to be little difference in richness and diversity. Functional homogenization of life history traits is often indicative of a highly disturbed landscape, in which a percentage of functional diversity loss has already occurred (Mangels et al., 2017). The functional similarities between broadleaf and plantation sites surveyed in this study suggests that the remnant patches of broadleaf woodland maintained within the plantation landscape may also be highly disturbed, but that this disturbance is dependent on the surrounding matrix. Using species richness measures alone would fail to detect this homogenization of functional diversity in broadleaf sites, therefore underestimating the potential impact of plantation forestry on lepidopteran functional diversity.

This is important for habitat management; simply assessing species richness and diversity without considering functional diversity may lead habitat managers concentrating on local factors, and in our study system providing somewhat misleading findings regarding the impact of surrounding disturbance in broadleaf sites compared to plantation sites. Our analysis suggests that lepidopteran communities in our study area are influenced more by landscape rather than local scale factors, even when considering naïve species richness. Moth communities in plantation dominated landscapes may reflect historical deforestation pressure, resulting in the persistence of species which are relatively tolerant of disturbance in both plantations and the remaining remnants of broadleaf woodland. Higher functional redundancy in broadleaf woodland emphasizes the importance of preserving native broadleaf woodland in anthropogenically impacted landscapes, to protect ecosystem health and functioning. However, simply preserving remnant patches of broadleaf is not sufficient to maintain moth species richness and functional diversity in plantation dominated landscapes, rather the complete landscape should be considered in order to preserve high naïve and functional species richness and diversity. This study provides evidence that lepidopteran populations do appear to be reduced in non‐native commercial plantation landscapes, but future research is necessary to determine the drivers of functional diversity loss.

## AUTHOR'S CONTRIBUTIONS

LK and KP conceived the idea and designed the methodology. SM designed the functional diversity indices and authored the R package used in analysis. Data collection and statistical analysis was carried out by LK. LK led the writing of the manuscript, with contributions from SM. All authors contributed critically to the drafts and gave final approval for publication.

## DATA ACCESSIBILITY

The data used in for this analysis is provided in the Supporting Information (Data S1).
