# Supplementary material for: The metric matters when assessing diversity: Assessing lepidopteran species richness and diversity in two habitats under different disturbance regimes
Source: Ecol Evol. 2018 Oct 26;8(22):11134–42. doi: 10.1002/ece3.4581 (PMC6262917; doi:10.1002/ece3.4581)
Supplement: Supplementary file 1 [file ECE3-8-11134-s001.docx]

# Appendix


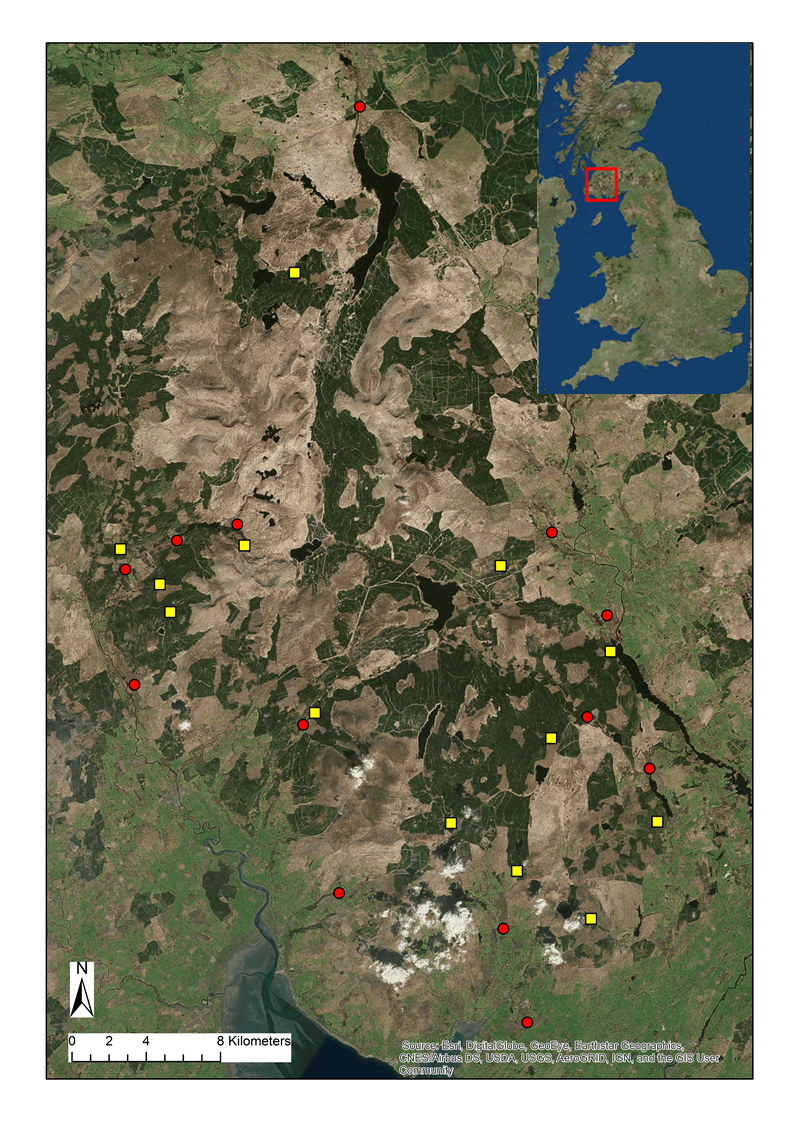


Figure 1. Map showing sites (broadleaf woodlands are displayed as yellow squares, plantations as red circles) and distribution around Galloway Forest Park.

Table 1: Description of constraints used in analysis.

| Measure | Description |  |  |  |
| --- | --- | --- | --- | --- |
| Naïve | Equal similarity matrix is provided, assumes that all species are equally different | | | |
| Taxonomic | Distance matrix describing differences in genera, family or suborder | | | |
| Host Plant | Difference in larval host plant preferences. Species categorised as preferentially foraging on the following plant groups: Bracken, coniferous trees, deciduous trees, detritus, flowering plants, grasses, moss and lichen, shrubs and trees, both deciduous and coniferous trees, generalist | | | |
| Larval Specialism | Difference in larval specialisms based on habitat preferences. Species categorised as follows: Herb generalist, herb specialist, Lichen specialist, lichen and wood specialist, wood generalist or wood specialist | | | |
| Overwintering Stage | Difference in overwintering stage between moth species. Species categorised as follows: Cocoon, egg, larva, pupa, various, na | | | |
| Wing Span | Moth wing span as reported from the literature. Average wing span 32.8 (15.0 - 77.5) mm | | | |

Table 2A: Example of naïve similarity matrix (all species are considered equally different from each other)

|  | *Cerapteryx graminis* | *Eugnorisma glareosa* | *Diarsia dahlii* | *Hylaea fasciaria* | *Eulithis pyraliata* | *Plagodis pulveraria* | *Mesoleuca albicillata* | *Autographa pulchrina* | *Epione repandaria* |
| --- | --- | --- | --- | --- | --- | --- | --- | --- | --- |
| *Cerapteryx graminis* | 0.00 | 1.00 | 1.00 | 1.00 | 1.00 | 1.00 | 1.00 | 1.00 | 1.00 |
| *Eugnorisma glareosa* | 1.00 | 0.00 | 1.00 | 1.00 | 1.00 | 1.00 | 1.00 | 1.00 | 1.00 |
| *Diarsia dahlii* | 1.00 | 1.00 | 0.00 | 1.00 | 1.00 | 1.00 | 1.00 | 1.00 | 1.00 |
| *Hylaea fasciaria* | 1.00 | 1.00 | 1.00 | 0.00 | 1.00 | 1.00 | 1.00 | 1.00 | 1.00 |
| *Eulithis pyraliata* | 1.00 | 1.00 | 1.00 | 1.00 | 0.00 | 1.00 | 1.00 | 1.00 | 1.00 |
| *Plagodis pulveraria* | 1.00 | 1.00 | 1.00 | 1.00 | 1.00 | 0.00 | 1.00 | 1.00 | 1.00 |
| *Mesoleuca albicillata* | 1.00 | 1.00 | 1.00 | 1.00 | 1.00 | 1.00 | 0.00 | 1.00 | 1.00 |
| *Autographa pulchrina* | 1.00 | 1.00 | 1.00 | 1.00 | 1.00 | 1.00 | 1.00 | 0.00 | 1.00 |
| *Epione repandaria* | 1.00 | 1.00 | 1.00 | 1.00 | 1.00 | 1.00 | 1.00 | 1.00 | 0.00 |

Table 2B: Example of taxonomic constrained similarity matrix

|  | *Cerapteryx graminis* | *Eugnorisma glareosa* | *Diarsia dahlii* | | *Hylaea fasciaria* | | *Eulithis pyraliata* | *Plagodis pulveraria* | *Mesoleuca albicillata* | *Autographa pulchrina* | *Epione repandaria* |
| --- | --- | --- | --- | --- | --- | --- | --- | --- | --- | --- | --- |
| *Cerapteryx graminis* | 0.00 | 1.00 | 1.00 | 1.00 | | 0.33 | | 1.00 | 1.00 | 1.00 | 0.33 |
| *Eugnorisma glareosa* | 1.00 | 0.00 | 0.33 | 0.67 | | 0.33 | | 0.67 | 0.33 | 0.33 | 1.00 |
| *Diarsia dahlii* | 1.00 | 0.33 | 0.00 | 0.33 | | 0.33 | | 1.00 | 0.33 | 0.33 | 0.33 |
| *Hylaea fasciaria* | 1.00 | 0.67 | 0.33 | 0.00 | | 0.33 | | 0.33 | 0.33 | 0.33 | 1.00 |
| *Eulithis pyraliata* | 0.33 | 0.33 | 0.33 | 0.33 | | 0.00 | | 1.00 | 1.00 | 0.67 | 1.00 |
| *Plagodis pulveraria* | 1.00 | 0.67 | 1.00 | 0.33 | | 1.00 | | 0.00 | 0.67 | 1.00 | 1.00 |
| *Mesoleuca albicillata* | 1.00 | 0.33 | 0.33 | 0.33 | | 1.00 | | 0.67 | 0.00 | 1.00 | 0.33 |
| *Autographa pulchrina* | 1.00 | 0.33 | 0.33 | 0.33 | | 0.67 | | 1.00 | 1.00 | 0.00 | 1.00 |
| *Epione repandaria* | 0.33 | 1.00 | 0.33 | 1.00 | | 1.00 | | 1.00 | 0.33 | 1.00 | 0.00 |
|  |  |  |  |  | |  | |  |  |  |  |

Table 2C: Example of functional constrained similarity matrix

|  | *Cerapteryx graminis* | *Eugnorisma glareosa* | *Diarsia dahlii* | *Hylaea fasciaria* | *Eulithis pyraliata* | *Plagodis pulveraria* | *Mesoleuca albicillata* | *Autographa pulchrina* | *Epione repandaria* |
| --- | --- | --- | --- | --- | --- | --- | --- | --- | --- |
| *Cerapteryx graminis* | 0.00 | 0.59 | 0.59 | 1.00 | 1.00 | 1.00 | 1.00 | 0.59 | 1.00 |
| *Eugnorisma glareosa* | 0.59 | 0.00 | 0.35 | 1.00 | 1.00 | 1.00 | 1.00 | 0.59 | 1.00 |
| *Diarsia dahlii* | 0.59 | 0.35 | 0.00 | 1.00 | 1.00 | 1.00 | 1.00 | 0.59 | 1.00 |
| *Hylaea fasciaria* | 1.00 | 1.00 | 1.00 | 0.00 | 0.59 | 0.35 | 0.59 | 1.00 | 0.35 |
| *Eulithis pyraliata* | 1.00 | 1.00 | 1.00 | 0.59 | 0.00 | 0.59 | 0.35 | 1.00 | 0.59 |
| *Plagodis pulveraria* | 1.00 | 1.00 | 1.00 | 0.35 | 0.59 | 0.00 | 0.59 | 1.00 | 0.35 |
| *Mesoleuca albicillata* | 1.00 | 1.00 | 1.00 | 0.59 | 0.35 | 0.59 | 0.00 | 1.00 | 0.59 |
| *Autographa pulchrina* | 0.59 | 0.59 | 0.59 | 1.00 | 1.00 | 1.00 | 1.00 | 0.00 | 1.00 |
| *Epione repandaria* | 1.00 | 1.00 | 1.00 | 0.35 | 0.59 | 0.35 | 0.59 | 1.00 | 0.00 |
